# Identification and validation of a novel candidate gene regulating net meat weight in Simmental beef cattle based on imputed next‐generation sequencing

**DOI:** 10.1111/cpr.12870

**Published:** 2020-07-28

**Authors:** Farhad Bordbar, Just Jensen, Min Du, Adam Abied, Wei Guo, Lingyang Xu, Huijiang Gao, Lupei Zhang, Junya Li

**Affiliations:** ^1^ Key Laboratory of Animal Genetics Breeding and Reproduction Ministry of Agriculture and Rural Affairs Institute of Animal Sciences Chinese Academy of Agricultural Sciences Beijing China; ^2^ Center for Quantitative Genetics and Genomics Aarhus University Aarhus Denmark; ^3^ Department of Animal Sciences Washington Center for Muscle Biology Washington State University Pullman WA USA; ^4^ Animal Genetic Breeding and Reproduction Institute of Animal Science Chinese Academy of Agricultural Sciences Beijing China; ^5^ Meat Science and Muscle Biology, Animal and Diary Science University of Wisconsin‐Madison Madison USA

**Keywords:** Differentiation, GWAS, NGS, NMW, Proliferation

## Abstract

**Objectives:**

Genome‐wide association studies (GWAS) represent a powerful approach to detecting candidate genes for economically important traits in livestock. Our aim was to identify promising candidate muscle development genes that affect net meat weight (NMW) and validate these candidate genes in cattle.

**Materials and methods:**

Using a next‐generation sequencing (NGS) dataset, we applied ~ 12 million imputed single nucleotide polymorphisms (SNPs) from 1,252 Simmental cattle to detect genes influencing net meat yield by way of a linear mixed model method. Haplotype and linkage disequilibrium (LD) blocks were employed to augment support for identified genes. To investigate the role of *MTPN* in bovine muscle development, we isolated myoblasts from the longissimus dorsi of a bovine foetus and treated the cells during proliferation, differentiation and hypertrophy.

**Results:**

We identified one SNP *(rs100670823*) that exceeded our stringent significance threshold (*P* = 8.58 × 10^−8^) for a putative NMW‐related quantitative trait locus (QTL). We identified a promising candidate gene, *myotrophin* (*MTPN*), in the region around this SNP *Myotrophin* had a stimulatory effect on six muscle‐related markers that regulate differentiation and myoblast fusion. During hypertrophy, *myotrophin* promoted myotube hypertrophy and increased myotube diameters. Cell viability assay and flow cytometry showed that *myotrophin* inhibited myoblast proliferation.

**Conclusions:**

The present experiments showed that *myotrophin* increases differentiation and hypertrophy of skeletal muscle cells, while inhibiting their proliferation. Our examination of GWAS results with in vitro biological studies provides new information regarding the potential application of *myotrophin* to increase meat yields in cattle and helpful information for further studies.

## Graphical abstract

Myotrophin (MTPN) found associated with bovine net meat weight (NMW) trait by genome‐wide association studies (GWAS), inhibited bovine myoblasts proliferation and enhanced myogenic differentiation and hypertrophy.

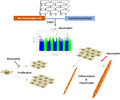

## 1 INTRODUCTION

Domesticated animals, especially cattle, are recognized for their economic importance in countries around the world.^1^ Cattle breeds have undergone organized selection to enhance beef production^2^ according to breeding agendas based on meat‐related traits.^3^ Beef breeders prefer rapid lean muscle mass growth to meet dramatically increasing consumer demands for lean meat.^4^ Hence, muscle tissue growth characteristics are economically very important.

Skeletal muscle development in the foetal stage is crucial because there is no net increase in muscle fibre number after birth. Foetal skeletal muscle development involves myogenesis, adipogenesis and fibrogenesis, which are all produced by mesenchymal stem cells.^5^ Skeletal muscle growth is achieved by an increase in myofibre number (hyperplasia) and size (hypertrophy). Hypertrophy, generally defined as an expansion of myotube size, is the main determinant of skeletal muscle mass.^6^ During myogenesis, myogenic regulatory factors play critical roles in the progression of differentiation. The actuation of these factors, including *MyoD* and *MyoG*, activate expression of myosin heavy chain (*MyHC*), a tremendously important protein in myotube formation.^7^ Isoforms of the *MyHC* gene (eg *MYH1*, *MYH2*, *MYH3* and *MYH4*) can be used as fusion markers, providing key information about differentiation in myogenesis. *MyHC* isoforms also represent a robust tool for characterizing muscle fibre types in skeletal muscle.^8^ *MYH1* and *MYH4* are typically expressed in fast muscle fibres,^9^ whereas *MYH3* is expressed preferentially in slow muscle fibres.^10^ Hence, identifying genes that can upregulate myoblast differentiation and hypertrophy can provide a benefit for beef cattle production.

The Simmental breed of cattle is one of the oldest and most widespread cattle breeds in almost every region of the world. This breed is typically bred for fast‐growing performance and lean meat production under a proper feeding regimen.^11^, ^12^, ^13^ Many studies have examined Simmental cattle in terms of economically important traits, such as growth,^14^ carcass quality,^15^ meat quality^16^ and meat yield.^17^ A better understanding of the genetic variation in meat quality and yield will help guide breeders to adopt methods that enable beef market demands to be efficiently addressed.

As meat production in the beef industry has grown considerably, breeders have become increasingly focused on pinpointing quantitative trait loci (QTLs) and candidate genes that may affect cattle growth and meat production traits. Genes associated with muscle characteristic and development are distributed over many chromosomes, with relevant QTLs identified on bovine chromosomes 2, 3, 4, 6, 20 and 29.^18^, ^19^, ^20^, ^21^, ^22^, ^23^

Genome‐wide association studies (GWAS) employing high‐density SNP panels represent a powerful approach to detecting regions of the genome and genetic variants that can explain variation in complex disorders and clinically important traits in humans^24^, ^25^, ^26^ and domesticated animals.^19^, ^27^, ^28^, ^29^ GWAS outputs are sensitive to several factors, including sample size and the number of variants influencing a target trait.^30^ Next‐generation sequencing (NGS) can be used to identify many more genetic variants than are used in association studies employing SNP arrays. Sharma et al used NGS to localize 18 putative variants related to Mendelian diseases in Hanwoo cattle,^31^ and another 33 genes related to domestication.^32^ This method provides a robust strategy with which to explore genes with important influences on complex traits. In Simmental beef cattle, NGS has revealed several genes that regulate the dimensions of the hind quarters, including *SLC13A1, LMOD2*, *WASL, IQUB*, *NDUFA5, ASB15* and *PLXNA4*.^18^ However, the high densities of SNPs in NGS datasets complicate quantification of marker effects.^18^, ^33^

Moreover, a shortcoming of GWAS is errant rejections of the null hypothesis, leading to many false positives.^34^ Hence, GWAS results must be validated before such findings can be applied for selection of the traits of interest as well as for confirming gene function and verifying the biological significance of detected genes.^34^, ^35^

Net meat weight (NMW, carcass weight without bone) has become an important parameter in the meat industry because of its direct correlation with other economically important traits, such as live weight and carcass weight.^36^ The objectives of this research were firstly to conduct a GWAS with an imputed NGS dataset aimed at detecting NMW candidate genes in Simmental beef cattle and, secondly, to validate the functions of identified genes appearing to affect both the differentiation and proliferation of myoblasts. This line of research is important for breeding programmes because it provides comprehensive knowledge and confirmation of the associations. Ultimately, our long‐term aim is to provide validated information for useful candidate genes to help Simmental breeders select for breeding animals that will yield offspring with increasing meat yields for consumers.

## 2 MATERIALS AND METHODS

### 2.1 Animal resources and phenotype data

Our Simmental beef cattle study population consisted of 1,346 individuals born between 2009 and 2015. Originally, the cattle were obtained from Ulgai, Xilingol League and Inner Mongolia in China. After weaning, they were taken to Jinweifuren farm feedlot in Beijing. All calves were raised in accordance with standardized conditions (they were fed with a total mixed ration (TMR) in accordance with the eighth revised edition of nutrition requirement of beef cattle (NRC, 2018)). Animals were slaughtered at 16‐18 months of age with electrical stunning followed by bloodletting. Immediately after bloodletting, carcasses were cut open vertically to remove internal organs and hide. Then, hot carcass weights were determined by subtracting the weight of the head, internal organs and hide organs from the weight of the whole non‐chilled carcass weight. The carcasses were stored at 4°C. NMW was determined by subtracting the bone weight from the hot carcass weight. Mean NMW value, standard deviation, maximum and minimum were 231.77 kg, 41.4 kg, 395 kg and 126 kg respectively.

### 2.2 Genotype analyses and quality control

Illumina Bovine HD SNP Beadchip (770k) and genotype analyses were performed in Illumina Genome Studio (Illumina, SD, CA, USA). We conducted quality control by withdrawing animals with high Pi‐Hat values (Showing duplication of sample). The following strict criteria for excluding SNPs and animals were applied: SNP call rate < 90%, minor allele frequency < 5%, Hardy‐Weinberg equilibrium deviation *P* < 10^−6^, and > 10% animals missing genotype data. These criteria were tested in PLINK v1.07 software.^37^ Following exclusion of 94 animals, a final cohort of 1,252 cattle with 671,204 autosomal SNPs were included in subsequent analyses.

### 2.3 Resequencing

In accordance with Pi‐Hat values and genomic relationships, 44 unrelated animals were selected for resequencing. Genomic DNA was extracted using a TIANamp Blood DNA kit (Tiangen Biotech Company Limited, BJ, CHN). DNA concentration and purity were measured with a NanoPhotometer N50 (Implen, MU, DE). 1.5 μg genomic DNA with an A260/280‐nm absorbance ratio of 1.8‐2.0, and A260/230‐nm absorbance ratio of 2.0‐2.2 was fragmented using a Covaris Ultrasonicator S2 (Covaris, Woburn, MA, USA). Sequencing libraries were constructed with the Truseq Nano DNA HT sample preparation kit (Illumina Inc, SD, CA, USA) according to the manufacturer's instructions. Each sample was given a sequence‐recognition index code. The DNA samples were fragmented into ≤ 350‐base pair fragments with sonication, and the DNA fragments were subjected to end polishing, A‐tailing addition and exposure to a full‐length adapter for sequencing with subsequent PCR amplification. Sequence libraries for 44 individual animals (44 libraries) were run in an Illumina Hiseq 2500 genome sequencing system (Illumina Inc, San Diego, CA, USA). A total of 9,621,765,847 reads were acquired, in which poor‐quality reads with > 10% undiscovered bases, >10% mismatches or > 50% low‐quality bases were excluded. We removed duplicates from PCR amplification readouts in the formation of our library. The samples had an average sequencing depth of ~ 20× (quantity of sequences for each base).

### 2.4 Imputation of SNP

Sequences with a minor allele frequency > 0.05 were imputed. A total of 21,043,398 sequence variants from the 44 genetically sequenced animals were analysed in BEAGLE v4.1,^38^ (default setting) with algorithms determined by population data to deduce genotypes for animals with missing information and haplotypes. For imputed sequence variants, genotypes were labelled as 0, 1 or 2 for homozygotes, heterozygotes and alternative homozygotes, respectively. Ultimately, we retained 12,468,401 SNPs for chromosomes 1‐29 from the RNA sequencing data applying the key criterion of imputation quality > 0.1.^39^

### 2.5 Statistical model

A general mixed linear model for NMW was developed according to the formula $y=\mu +Xb+m_{j}b_{j}+Zu+e$, wherein *y* represents a phenotypic value and *μ* is a population mean. Two fixed effect variables based on single marker regression were applied, including *b*, representing noise related to fixed effects (gender, weight, birth year and fattening days), and *b_j_*, representing the effect of an SNP. The parameter *m_j_* represents the vector for the *i^th^* marker, *u* is the polygenic effect presumed with N* (0, σ* ^2^ *K)*, and *K* corresponds to the kinship matrix. Although all SNPs on autosomal chromosomes were eligible for inclusion, those SNPs on the chromosomes where *m_j_* resided, were excluded. The *σ* ^2^ parameter represents additive genetic variance, *X* represents the incidence matrix by which phenotypic values relate to fixed effects, and *Z* represents the matrix by which phenotypic values relate to polygenic effects. Finally, the variable *e* represents random residual effects, presumed in the formula ${V(e)=I\sigma }_{e}^{2}$, wherein I is the identity matrix and $\sigma_{e}^{2}$ is the residual variance.

Associated SNPs were detected with GenABEL v1.8‐0,^40^ in R software, applying a significance criterion of *P* < .05 with Bonferroni correction on the basis of dividing by the effective number of SNPs. Dependable QTLs and putative candidate genes were found with the following actual significance thresholds: segment 1, *P* = 8.58 × 10^−8^; segment 2*, P* = 1.24 × 10^−8^; and segment 3, *P* = 1.95 × 10^−8^. To ascertain each SNP *p* values, t statistics were determined. Our candidate gene was detected with the UCSC genome browser (http://www.genome.ucsc.edu). Using PLINK v1.07,^37^ we constructed haplotype blocks *(plink‐‐bfile mydata‐‐blocks)* to estimate linkage disequilibrium (LD) for SNPs ≤ 200 kb in length. The *plink‐‐bfile mydata‐‐hap plink*.*blocks‐‐hap‐freq* commands^37^ were used to analyse haplotype associations in each block. Haplotype blocks for each chromosome were visualized in Haploview v4.2 software.^41^

### 2.6 SNP propagation

Our NGS dataset included a massive quantity of high throughput markers, which can complicate SNP *p*‐value determination by conventional methods.^18^, ^33^ We dealt with this problem by segregating chromosomes into segments: segment 1, chromosomes 1‐10 with 5,830,727 SNPs; segment 2, chromosomes 11‐20 with 4,063,690 SNPs; and segment 3, chromosomes 21‐29 with 2,573,984 SNPs. Accordingly, segment‐specific *p*‐value thresholds were used depending on the number of markers in each segment.

### 2.7 Primary cell isolation, cell culture, *MTPN* treatments for differentiation and hypertrophy

The *longissimus dorsi* (300 mg) was removed from foetuses, washed with phosphate‐buffered saline (PBS) and diced into small pieces. The fragments were digested with Dulbecco's modified Eagle's medium (DMEM, Gibco, Grand Island, NY, USA) with 0.1% collagenase type IV (Sigma, MO, TX, USA) for 45 min on at shaker at 37°C. The medium mixture was filtered through the 40‐μm diameter nylon meshes, then supplemented with growth medium (GM) consisting of DMEM with 10% foetal bovine serum (Gibco, Grand Island, NY, USA) and centrifuged. The cells were resuspended in GM and seeded in petri dishes. One day later following attachment of muscle cells, the GM was replaced to remove dead cells. Cells were subcultured with trypsin (Amresco, MO, TX, USA) when they reached 75% confluency. Subsequently, we cultured the cells in 12‐well plates with GM. When cells reached 100% confluence, GM was changed to differentiation medium (DM) consisting of DMEM supplemented with 5% horse serum, and this day was considered to be day 0.

**Figure 1:**
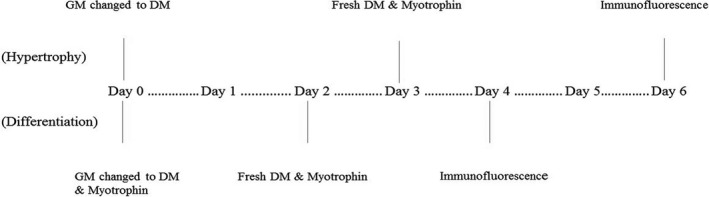
In vitro differentiation and hypertrophy induction study design

Recombinant bovine *myotrophin MTPN* (Cusabio, HO, TX, USA) was reconstituted in sterilized water. *Myotrophin* (10 ng/mL, 50 ng/mL, 200 ng/mL or 1000 ng/mL) was applied to ascertain dose‐related efficiency. As shown in Figure 1, *myotrophin* was supplied on days 0‐4 for differentiation and supplied on days 3‐6 for hypertrophy.

### 2.8 RNA isolation, reverse transcription and quantitative real‐time PCR (qRT‐PCR)

RNA was extracted from cells with TRIzol reagent (Invitrogen, Carlsbad, CA, USA). RNA concentration and purity were monitored with a NanoPhotometer N50 (Implen, MU, DE); contamination was determined by 1.5% agarose gel electrophoresis. From 500 ng RNA, First‐strand cDNAs were synthesized with PrimeScript RT Master Mix (TaKaRa, Kusatsu, JPN).^42^ We designed forward and reverse primers in Primer Premier 5.0 software (Premier Biosoft International, CA, USA) (Table S1). To investigate the effect of *myotrophin* on the expression of muscle‐related genes (*MyoD*, *MyoG*, *MYH1*, *MYH2*, *MYH3* and *MYH4*), qRT‐PCR was conducted in a QuantStudio 7 Flex real‐time PCR system (Life, Carlsbad, CA, USA) with *18s* as an internal reference gene.

### 2.9 Immunofluorescence

Myotubes were fixed with 3‐4% paraformaldehyde in PBS, washed three times with cold PBS^43^ and then incubated for 10 min in PBS containing 0.1% Triton X‐100. After three subsequent 5‐min washes with PBS, cells were blocked for 35 min in blocking solution containing 1% albumin bovine serum (Beyotime, SH, CHN). Myotubes were incubated overnight at 4°C with anti‐myosin heavy chain (*MyHC*) antibody (1:100, Developmental Studies Hybridoma Bank, IA, USA). Following washing, they were treated with FITC‐labelled goat anti‐mouse IgG (1:1000, Beyotime, SH, CHN) for 1 h at room temperature in the dark. Nuclei were counterstained with 4′,6‐diamidino‐2‐phenylindole dihydrochloride (DAPI, Sigma‐Aldrich, MO, USA) for 1 min. Images were acquired and viewed with a fluorescence microscope (TCS SP8, Leica, DE).

### 2.10 Fusion index and myotube diameter

For each coverslip, five locations dispersed across each construct were imaged. Calculations of the number of nuclei were performed in ImageJ software.^44^ Myotubes were identified by positive *MyHC* staining. The number of nuclei in myotubes was determined by counting the number of nuclei that co‐localized with positive *MyHC* staining. The myogenic fusion index was calculated by dividing the number of nuclei in myotubes by the total number of nuclei. The fusion index was determined as the ratio of the number of nuclei in *MyHC*‐positive myotubes to all nuclei scattered in five random fields. Myotube diameter was analysed in Image Pro Plus 6.0 software (Media Cybernetics, MD, USA). Up to four representative measurements of myotube width were collected in each image, depending on the number of myotubes present in the image.

### 2.11 CCK assay

Upon reaching 75% confluency, cells were subcultured and seeded in 96‐well plates at a density of 3000 cells per well with different *myotrophin's* concentrations. Following incubation for 0 h, 24 h, 48 h, 72 h and 96 h at 37°C, cell viability was determined by CCK‐8 assays performed according to the manufacturer's instructions. In brief, 10 µl of CCK‐8 reagent (Beyotime, SH, CHN) was added to each well and cells were incubated at 37°C in 5% CO_2_ for 2 h. Absorbance was determined at a wavelength of 450 nm with an Imark Microplate Reader (Bio‐Rad, CA, USA). Each assay was performed in triplicate and repeated three times.

### 2.12 Flow cytometry

Cells (6 × 10^4^ cells/cm^2^) were seeded with experimentally indicated *myotrophin* concentrations and cultured in GM at 37°C in 5% CO_2_. After 72 h, the cells were trypsinized and transferred into 15‐mL centrifuge tubes with fresh medium. Approximately 10^6^ cells were centrifuged for each concentration. After resuspending the cells in 500 μL PBS, they were fixed in 4.5 mL of 70% cold ethanol for 3 h, centrifuged for 5 min at 200 × *g*, and washed with 5 mL PBS. The cells were stained with 1 mL of propidium iodide (PI, Beyotime, SH, CHN) solution for 30 min at room temperature. Cell cycle analysis was performed in a FACS Calibur machine with Modfit (Verity Software House, ME, USA).^45^

### 2.13 Statistical analysis

The data were subjected to one‐way and two‐way analyses of variance (ANOVAs) with Tukey's post hoc tests. For each experiment, at least three replicates were considered. Statistical analysis was executed in GraphPad Prism (version 6.0, GraphPad Software, San Diego, CA, USA) with a significance criterion of *P* < .05.

## 3 RESULTS

### 3.1 Association analysis revealed MTPN as a potential candidate gene of the NMW trait

**Figure 2:**
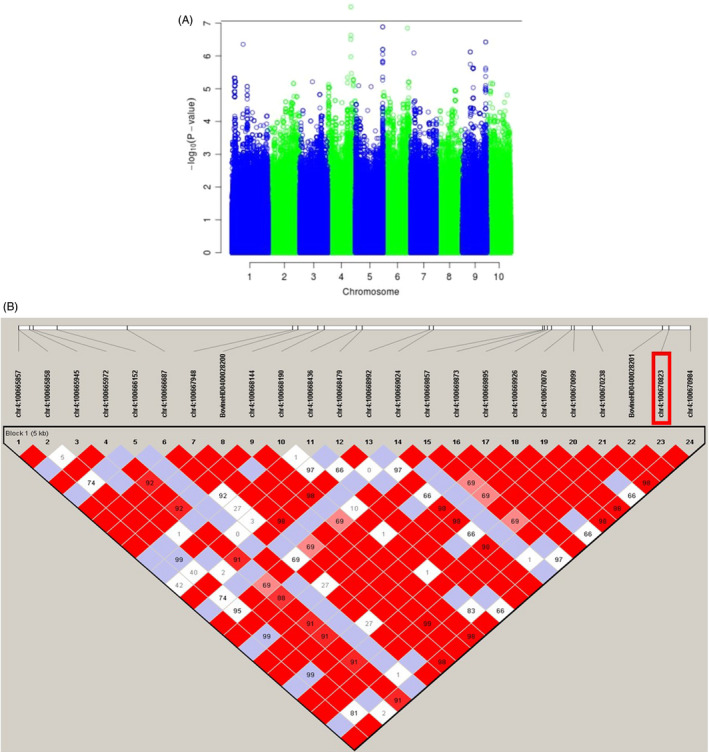
Manhattan plot of ‐log_10_ (p) for the NMW‐associated segment. A, the threshold is illustrated by a horizontal line. B, LD plot showing a 5‐kb high‐LD block harbouring the most associated SNP in segment 1 of bovine chromosome 4

Of 9,621,765,847 raw reads obtained, our quality control processes retained 9,584,920,309 reads. The quality of the sequencing data was excellent (Q20 ≥ 94.84% and Q30 ≥ 88.71%).^46^ In segment 1, we identified one significant SNP on bovine chromosome 4 (*rs100670823*, *P* = 3.2 × 10^‐8^), which we listed as a candidate putative QTL related to NMW (see Manhattan plot for Segment 1 in Figure 2A). The marker *rs100670823* was located 44 kb upstream of the *MTPN* gene which was a very promising candidate gene for NMW trait influence. Haplotype studies illustrated that this SNP was placed in a 22‐kb haplotype block within a high‐LD 5‐kb span, as shown in Figure 2B. Remarkably, the amino acid sequences of MTPN were found to have 100% identity between cattle and human (Figure S1).

### 3.2 *MTPN* promoted differentiation of myoblasts into myotubes

**Figure 3:**
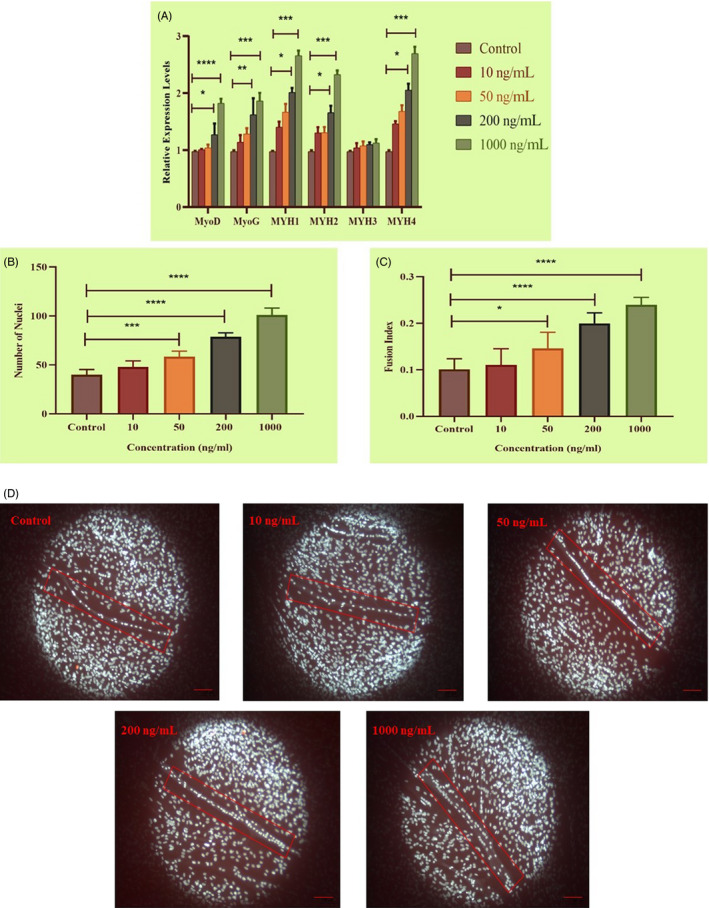
*MTPN* promotion of muscle differentiation evidenced by qRT‐PCR, number of nuclei and fusion index. A, Relative mRNA expression levels of *MyoD*, *MyoG*, *MYH1*, *MYH2*, *MYH3*, and *MYH4* assessed after addition of MTPN (in ng/mL: 10, 50, 200 and 1000) (*18s*, reference gene). B and C, DAPI‐stained nuclei revealing numbers of nuclei and calculated fusion index values. D, Representative fluorescence images of nuclei within myotubes at indicated concentrations. One‐way and two‐way ANOVAs with Tukey's post hoc testing*;* ****P < .0001, ***P < .001, **P < .01 and *P < .05

To investigate the effect of myotrophin on the differentiation of bovine foetus derived myoblasts, cells were treated with 0 ng/mL, 10 ng/mL, 50 ng/mL, 200 ng/mL and 1000 ng/mL *MTPN* during differentiation. qRT‐PCR experiment showed that 1000 ng/mL *MTPN* significantly upregulated the expression of the muscle‐specific genes *MyoD* and *MyoG* with almost twofold increase compared to control (*P <* .0001 for *MyoD*, *P <* .001 for *MyoG*), and induced more than twofold increase of *MYH1*, *MYH2* and *MYH4* expression compared to the control group (*P <* .001). Expression of *MYH3* was not altered significantly by *MTPN* treatment (Figure 3A). The number of nuclei per myotube, an index of the differentiation of skeletal muscle, was significantly enhanced by most of the tested concentrations of *MTPN*. Figure 3B, *(P <* .0001, *P* < .001 vs. control) indicated an increase in myotube formation. The 10 ng/mL concentration was ineffective in this regard. Fusion index, calculated at day 4 by counting the number of nuclei in myotubes and total nuclei, was increased significantly in the 50 ng/mL *MTPN* group (*P < *.05 vs. control; Figure 3C), in the 200 ng/mL and 1000 ng/mL groups (both *P <* .0001). The numbers of nuclei in myotubes after *MTPN* treatment are shown in Figure 3D. These data show that *MTPN* promoted myogenic differentiation in a dose‐dependent manner.

### 3.3 *MTPN* promoted myotube hypertrophy

**Figure 4:**
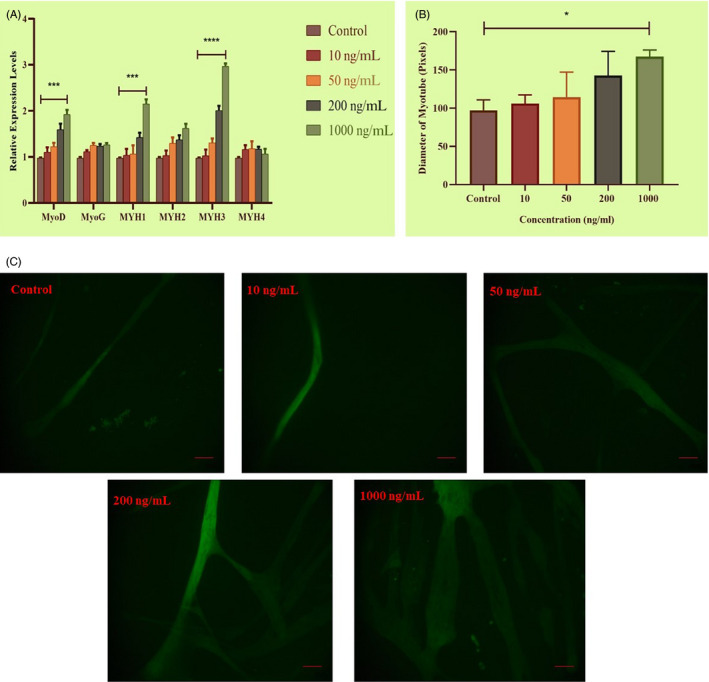
*MTPN*‐induced muscle hypertrophy. A, Relative mRNA expression of *MyoD*, *MyoG*, *MYH1*, *MYH2*, *MYH3* and *MYH4* assessed after addition of *MTPN* (in ng/mL: 10, 50, 200 and 1000) demonstrated by qRT‐PCR. B, Comparison of myotube diameters of *MTPN* treatment groups. C, Representative images showing the morphology of MyHC‐immunolabelled myotubes (green). One‐way and two‐way ANOVAs with Tukey's post hoc testing*;* ****P < .0001, ***P < .001, **P < .01 and *P < .05

As shown in Figure 4A, qRT‐PCR showed that *MTPN* treatment (1000 ng/mL) from day 3 onward increased expression of *MyoD*, *MYH1* and *MYH3* significantly (*P* < .001 for *MyoD* and *MYH1* genes with almost twofold increase compared to control; *P <* .0001 for *MYH3* with more than threefold increase compared to control). Analysis of *MyHC*‐immunolabelled myotubes showed that *MTPN* (1000 ng/mL) increased myotube diameter significantly (*P <* .05, Figure 4B,C). These results indicate that *MTPN* can have a positive effect on myotube hypertrophy in cattle.

### 3.4 *MTPN* attenuated skeletal muscle cell proliferation

**Figure 5:**
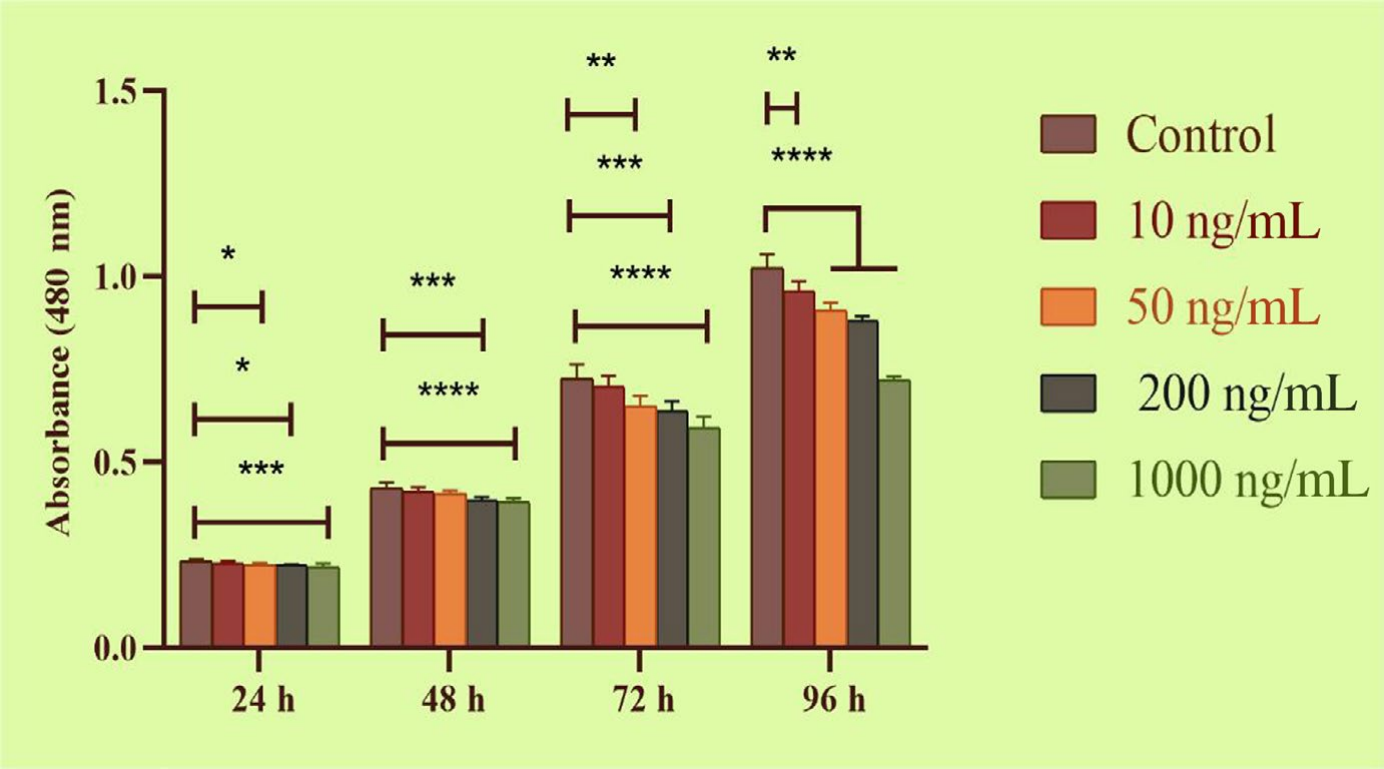
*MTPN* decreased proliferation of skeletal muscle cells, revealed in CCK8 assays, across four time points and four concentrations. One‐way and two‐way ANOVAs with Tukey's post hoc testing*;* ****P < .0001, ***P < .001, **P < .01 and *P < .05. Means ± standard deviations of six independent experiments are shown

As shown in Figure 5, CCK8 assays showed that 24‐h myoblast proliferation was inhibited by *MTPN* (*P <* .05 vs. control, 50 ng/mL and 200 ng/mL groups; *P* < .001 vs. control, 1000 ng/mL group). At 96 h, myoblast proliferation was suppressed in all *MTPN* treatment groups compared with the control group (*P <* .01, 10 ng/mL; *P* < .0001, 50 ng/mL, 200 ng/mL and 1000 ng/mL). These data show that *MTPN* inhibited myoblast proliferation in a dose‐dependent manner.

### 3.5 *MTPN* decreased the percentage of cells in S and G2/M phases

**Figure 6:**
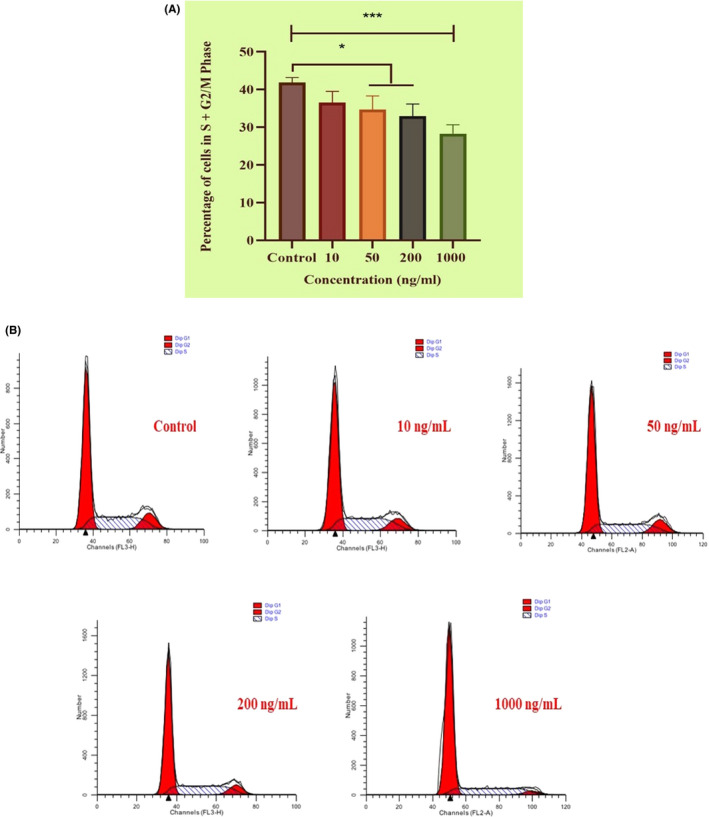
Flow cytometry data showing *MTPN*‐induced reductions in proliferating cell percentages. A, mean percentages (±SDs) of cells in S and G2/M phase following treatment with indicated experimental doses of *MTPN* from three independent experiments. B, Phase comparisons depicted by flow cytometry profiles. One‐way ANOVAs with Tukey's post hoc testing; ****P < .0001, *P < .05

Flow cytometry cell cycle analysis showed that myoblasts treated with 0 ng/mL (control), 10 ng/mL, 50 ng/mL, 200 ng/mL and 1000 ng/mL *MTPN* had, respectively, 41.80%, 39.87%, 38.19%, 35.91% and 26.23% proliferating cells in S and G2/M phase (Figure 6). The 1000 ng/mL concentration had the most dramatic reducing effect on cell proliferation (*P* < .001), followed by the 50 ng/mL and 200 ng/mL concentrations (both *P* < .05). The 10 ng/mL concentration did not affect proliferating cell levels significantly.

## 4 DISCUSSION

### 4.1 Biological function validation is important in post‐GWAS study

The application of NGS data in association studies has numerous advantages over low‐density genome variant studies for candidate gene detection, including higher throughput sequencing, more specific applicability, and higher read quality for sequencing dataset.^47^ There are a variety of validation methods that can be applied to GWAS results, such as high‐power statistical analyses (ie broad replication), genetic filtering (ie resequencing, deep sequencing, fine mapping), statistical filtering (ie genetic modelling, multiple loci, replication with heterogeneity) and phenome mapping,^34^ as well as studies using different populations^48^ and studies with population substructure corrections.^49^ Biological and functional validation, including molecular function, in vitro, and in vivo studies, is critical for supporting GWAS results^34^ and guiding the planning of follow‐up. In the present study, we attempted to verify an identified candidate gene in an in vitro functional study with proliferation and myogenic differentiation assays demonstrated to be reliable in vitro tools with which to investigate skeletal muscle development.^50^, ^51^, ^52^

### 4.2 Hypertrophy promotion

Skeletal muscle‐derived stem cells have shown a strong capacity for muscle growth, repair and regeneration. During these processes, muscle‐derived stem cells are regulated by various muscle‐specific factors.^7^ *MTPN* was identified originally in a hypertensive rat heart model and has been shown to regulate growth of myocardial cells.^53^ Notably, Anderson et al showed that recombinant human *MTPN* can induce cardiomyocyte hypertrophy and to increase myotrophin mRNA expression levels in human dilated cardiomyopathic hearts.^54^ Additionally, in a study examining *MTPN* localization and expression levels in pigs, Wang et al showed that MTPN is important for skeletal muscle growth and development.^55^ In a study investigating the effects of injected *MTPM* on the morphology and growth of skeletal muscle cells in mice, Shiraishi et al observed that, relative to control mice, *MTPN*‐treated mice had increased body weights and muscle fibres that were completely more regular morphologically, demonstrating *MTPN*’s muscle growth‐promoting activity in mice.^56^ However, in cattle, especially Simmental cattle, there has been a complete lack of information regarding *MTPN* and its effects on the growth and development of skeletal muscle as well as on the morphology of cells. In this study, after identifying *MTPN* as a candidate gene, we demonstrated positive effects of *MTPN* on skeletal muscle development, including hypertrophy, and myotube morphology. Myotube diameter was used as a parameter reflective of muscle hypertrophy.^57^ Using the same parameter of hypertrophy, Hayashi et al showed that MTPN and insulin‐like growth factor‐1 increase chick skeletal muscle hypertrophy.^58^ Our myotube diameter data affirm that *MTPN* is a positive growth factor on skeletal muscle cells in cattle.

### 4.3 Differentiation promotion

Subsequently, we investigated *MTPN* effects on myoblast differentiation, which has been rarely reported previously. To evaluate myoblast differentiation, we quantified average number of nuclei per myotube and calculated fusion index values, parameters that have been widely used for this purpose. Here, we determined mean numbers of nuclei per myotube and fusion index values to assess differentiation of skeletal muscle. Interestingly, our results revealed that different concentrations of *MTPN* had positive and promoting effects on the number of nuclei and the fusion index, leading us to conclude that it has a stimulating effect on differentiation.

It was shown that *S‐myotrophin* had stimulating effect on protein synthesis; such an effect was not seen with myoblast proliferation previously.^59^ Hayashi et al reported that *MTPN* played crucial anabolic impact on protein synthesis, without altering incorporation of H‐leucine into proliferating myoblasts, demonstrating that their data reflects an effect on hypertrophy rather than proliferation.^58^ Here, we used CCK‐8 proliferation assays to examine *MTPN* effects on proliferation and observed a rather surprisingly dose‐dependent attenuating effect on cell proliferation. Moreover, our flow cytometry cell cycle analysis indicated that *MTPN* decreased the percentage of cells in S and G2/M phase. These CCK‐8 assay and flow cytometry results led us to conclude that *MTPN* can act as a negative regulator of myoblast proliferation.

The MTPN pathway has been shown to be an important regulator of skeletal muscle hypertrophy, differentiation and proliferation. Activation of NF‐κB (nuclear factor kappa‐light‐chain‐enhancer of activated B cells) has been shown to be involved in the hypertrophic response to *MTPN* in neonatal rat ventricular cardiomyocytes, implicating the protein kinase C–IκB kinase–NF‐κB pathway in mediating the *MTPN*‐induced hypertrophic response in cardiomyocytes.^60^ Indeed, deficiency of classical NF‐κB signalling members enhances myogenic differentiation and alters myotube homeostasis.^61^ Lu et al reported that muscle‐derived stem cells isolated from the p65^±^ mice had enhanced proliferation and myogenic differentiation compared to those from wild‐type littermates.^62^ However, the exact mechanism by which *MTPN* regulates muscle development requires further investigation.

In our research, we identified *MTPN* as a promising candidate gene for NMW modulation and then verified the biological functionality of *MTPN* in an in vitro cell culture model. In light of the body of results obtained in this study, we have concluded that MTPN can increase myoblast differentiation and hypertrophy, while inhibiting muscle cell proliferation. These results suggest strongly that *MTPN* may be a key regulator of skeletal muscle growth and development in Simmental cattle. The present data thus provide useful information for meat industry science as well as helpful information for further studies.

## 5 ETHICAL APPROVAL AND CONSENT TO PARTICIPATE

All procedures were performed in accordance with regulations set by China's Council on Animal Care, and the study protocol was approved by the Institute of Animal Science at the Chinese Academy of Agricultural Science, Beijing, China. Animal analyses adhered to the principles of China's Council on Animal Welfare. Bovine foetuses were obtained rapidly at gestational ages of 90‐120 days by uterine dislodgment immediately after pregnant cows were slaughtered. Foetal cells were established in cultures within 2‐4 h of isolation.

## CONFLICT OF INTEREST

The authors declare that they have no competing interests.

## AUTHOR CONTRIBUTION

Farhad Bordbar was responsible for formal analysis, methodology, project administration, software, validation, visualization, writing – original draft and writing – review and editing. Just Jensen performed validation. Min Du and Wei Guo performed review and editing. Abeid Adam was responsible for data curation. Lingyang Xu and Huijiang Gao were responsible for conceptualization and project administration. Lupei Zhang and Junya Li were responsible for funding acquisition, project administration and supervision.

## Supporting information

Table S1

Fig S1

## Data Availability

The data underlying this study have been uploaded to Dryad. The raw genotype data are accessible using the following https://doi.org/10.5061/dryad.4qc06. Additional data are available using the https://doi.org/10.5061/dryad.5v3k1ct.
