# Supplementary material for: Identification and validation of a novel candidate gene regulating net meat weight in Simmental beef cattle based on imputed next‐generation sequencing
Source: Cell Prolif. 2020 Jul 28;53(9):e12870. doi: 10.1111/cpr.12870 (PMC7507581; doi:10.1111/cpr.12870)
Supplement: Supplementary file 1 — Table S1 [file CPR-53-e12870-s001.docx]

**Supporting Information (Table S1_SuppInfo)**

| **Gene names** | **Forward (5’-3’)** | **Reverse (5’-3’)** |
| --- | --- | --- |
| *18s* | GTAACCCGTTGAACCCCATT | CCATCCAATCGGTAGTAGCG |
| [*MyoD*](https://en.wikipedia.org/wiki/MyoD) | TTTGCCAGAGCAGGAGCCCCTC | TTCGAACACCTGAGCGAGCGC |
| *MyoG* | CAAATCCACTCCCTGAAA | GCATAGGAAGAGATGAACA |
| *MYH1* | GGGAAACTGGCTTCTGCTGAT | TGGGTTGGTGGTGATTAGGAG |
| *MYH2* | GTCAAAGGGACTATCCAGAGCAG | AGAAGAGGCCCGAGTAGGTGT |
| *MYH3* | TAAGGAAGAGTATGCCAAGGGG | CATCCAGGAGGTGTAGCGGTC |
| *MYH4* | CTCCTAATCACCACCAACCCATA | TGTCAGCAACTTCAGTGCCATC |

**Table S1. List of the forward and reverse primers used in quantitative real-time PCR (qRT-PCR).**
