# Supplementary material for: Identification and validation of a novel candidate gene regulating net meat weight in Simmental beef cattle based on imputed next‐generation sequencing
Source: Cell Prolif. 2020 Jul 28;53(9):e12870. doi: 10.1111/cpr.12870 (PMC7507581; doi:10.1111/cpr.12870)
Supplement: Supplementary file 2 — Fig S1 [file CPR-53-e12870-s002.docx]

**Supporting Information (Figure S1_SuppInfo)**

**
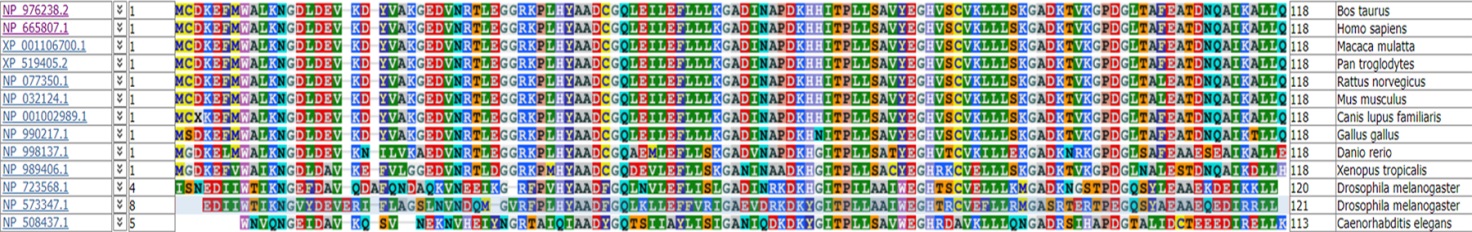
**

**Figure S1. Amino acid sequence identity of *MTPN* in different species shows that *MTPN* is conserved gene.** As shown, there is 100% identity between Amino acid sequences of *MTPN* in cattle and human.
